# Malignant granular cell tumor of chest wall: a case report

**DOI:** 10.3389/fonc.2024.1465130

**Published:** 2024-09-20

**Authors:** Fabiano Flauto, Alberto Servetto, Roberto Bianco, Luigi Formisano

**Affiliations:** Department of Clinical Medicine and Surgery, University of Naples Federico II, Naples, Italy

**Keywords:** rare tumor, skin tumor, granular cell tumor (Abrikosov tumor), recurrent granular cell tumor, multidiscipliary team

## Abstract

**Background:**

Granular Cell Tumors (GCTs), also known as Abrikossoff tumors, are rare neoplasms that typically originate from Schwann cells. These tumors most commonly occur in the head and neck region, particularly the tongue. While GCTs are generally benign, less than 2% of cases exhibit aggressive biological features such as rapid growth, high recurrence rates, and metastasis. In this report, we present a rare case of a Malignant Granular Cell Tumor (MGCT) of the chest wall, which posed significant challenges in both characterization and management.

**Case Presentation:**

A fifty-year-old man underwent an ultrasound examination for a nodular mass on his right chest wall. The ultrasound revealed a firm, hard mass measuring 2 cm x 2 cm with an uncertain diagnosis. A fine-needle aspiration biopsy (FNAB) guided by ultrasound was performed, resulting in a diagnosis of Abrikossoff tumor. The patient subsequently underwent radical excision of the mass, which confirmed the initial diagnosis. Nine months after surgery, a new mass with similar characteristics was detected during a physical examination. The patient underwent a second surgery, but this time the histopathological examination was negative for neoplastic cells. However, another mass appeared at the same site as the previous surgical excision. A CT scan and MRI of the right chest wall confirmed the presence of a 2 cm x 2 cm nodular mass. The patient then underwent a deeper excision. Histomorphological and immunohistochemical assessments confirmed the recurrence of MGCT.

**Conclusion:**

This case highlights the malignant potential of GCTs. The numerous local recurrences necessitated three surgeries and additional procedures. The aggressive nature of this pathology underscores the complexity of managing these tumors, which are poorly understood and lack proven post-operative strategies for controlling local and distant disease.

## Introduction

Granular Cell Tumors (GCTs) are rare, generally benign neoplasms of neural origin, most likely arising from Schwann cells (1). GCTs constitute approximately 0.5% of all soft tissue tumors and most commonly occur in adults aged 30 to 50, with a slight predominance in females. Although GCTs can develop in any part of the body, they are most frequently found in the head and neck region, particularly the tongue, which is the most common single site. Diagnosis of GCTs is primarily based on histopathological examination following a biopsy. The mainstay of treatment for GCTs is surgical excision with clear margins to prevent recurrence, and complete excision is generally curative for benign GCTs (2). Malignant GCTs, accounting for less than 2% of all GCTs, require more aggressive treatment, including wider surgical resection and may benefit from adjuvant radiotherapy or chemotherapy (3). This article aims to present an uncommon case of a chest wall MGCT and its complex, multidisciplinary management. We report this case in accordance with the CARE reporting checklist.

## Case presentation

A fifty-year-old man, who smoked fifteen cigarettes a day and had no significant medical history, presented with a firm, nodular mass on his right chest wall, in the submammary region. During the physical examination, a well-defined, hard-ligneous nodular area approximately 2 cm in diameter was detected on the right chest wall, inferior and lateral to the nipple-areola complex.

There was no ulceration or pigmentation of the overlying skin. There was no palpable peripheral lymphadenopathy. The patient had no systemic complaint. Routine hematological and biochemical investigations were normal.

**Figure 1:**
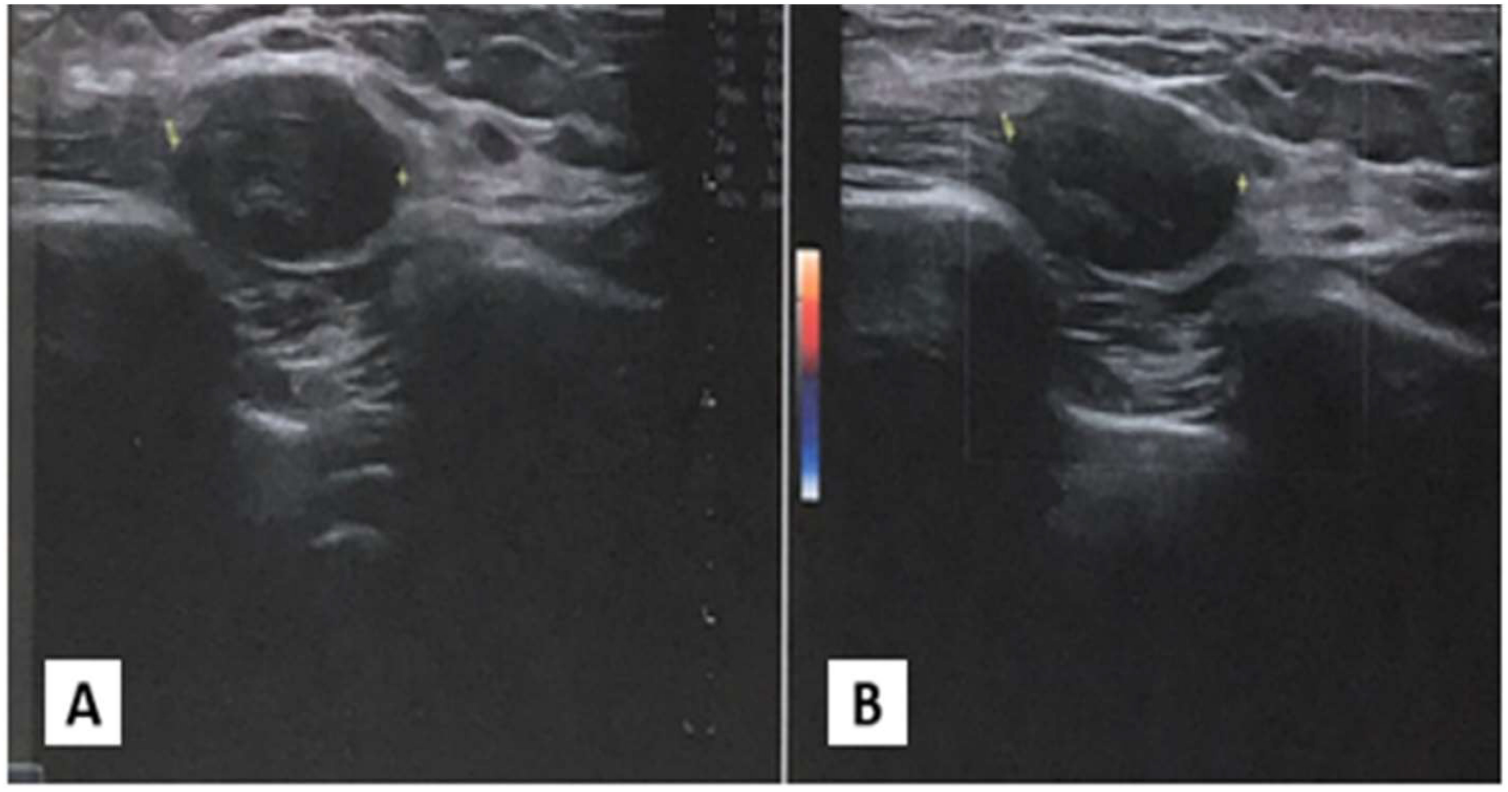
Ultrasonography of chest wall. **(A)** Ipoechoic soprafascial nodular mass with 2 cm x 2xm of diameter. **(B)** Absence of signal to Color Doppler examination.

An ultrasonographic evaluation of the right chest wall (**Figure 1**) revealed a hypoechogenic nodular mass with irregular margins, expanding in the subcutaneous plane and above fascial planes, measuring approximately 2 cm x 2 cm, with no signal on Color-Doppler analysis. No direct invasion was seen in the muscle or overlying skin. The differential diagnosis was very difficult, given the clinical features of the nodular mass.

In August 2022, an ultrasound-guided needle biopsy of the right chest wall, targeting the lower external quadrant of the breast, was performed, yielding a 1 cm tissue sample. The histopathologic diagnosis confirmed GCT. Immunohistochemical analysis revealed positivity for S100 and TFE3, and negativity for cytokeratins AE1/AE3, CD163, desmin, and MART1, with a Ki-67 proliferative index of 2%. These features were suggestive of GCT.

**Figure 2:**
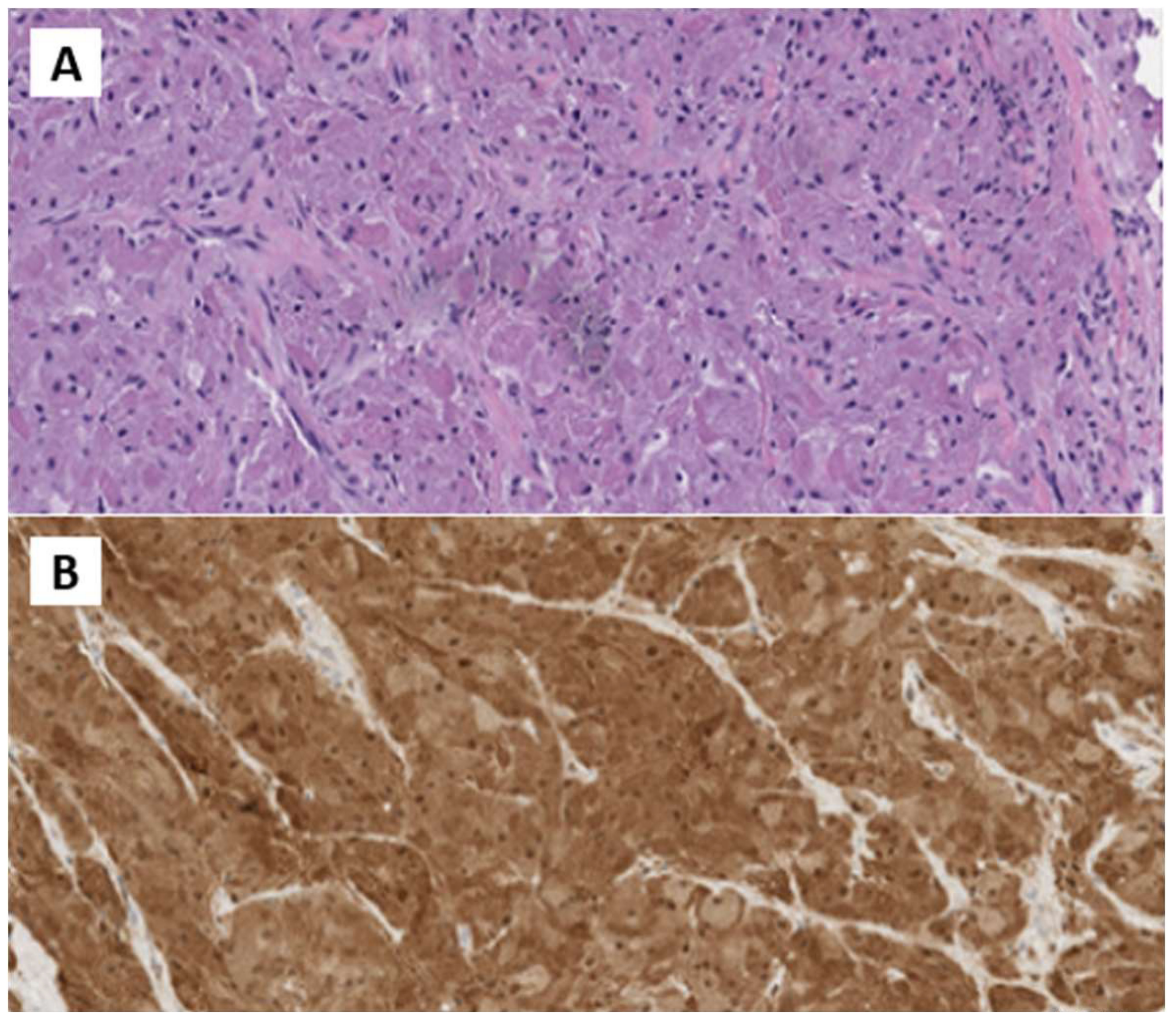
**(A)** H&E Medium power microphotographs demonstrating sheets or nests of large, polygonal tumor cells with abundant slightly basophilic and granular cytoplasm and vesicular nuclei infiltrating among skeletal muscle fibers. **(B)** Immunostained microphotograph showing that the tumor granular cells were positive for S-100 prote.

In October 2022, the patient underwent surgical excision. A 4 cm x 1.5 cm x 1.5 cm skin lozenge and a 3 cm x 2.5 cm fibro-yellowish nodular tissue were removed. The latter contained a nodular formation with a maximum diameter of 2 cm. Pathological examination showed fibroadipose and striated muscle tissue with neoplastic proliferation, exhibiting expansive and focally infiltrative margins. The lesion consisted of polygonal cells with eosinophilic cytoplasm and intracytoplasmic granules, organized in laminae. No necrosis or atypical mitoses were observed. The skin overlying the lesion was free from significant histopathological changes. Immunohistochemical analysis revealed positivity for S100 and CD68, and negativity for Cytokeratin PAN, with a Ki-67 index of 10%. The diagnosis was consistent with GCT with neoplastic infiltration of surgical margins (**Figure 2**).

In November 2022, a secondary surgical procedure was performed to enlarge the excision margins around the right breast region. The excised specimen measured 6 cm x 1 cm x 0.5 cm, including a central surgical scar of 4 cm. Pathological examination revealed dermal scar fibrosis with no residual neoplastic tissue.

Considering the unusual behavior of this neoplasm and the uncertainty of its histology, we requested a revision of the tissue samples from a center with high expertise in GCT and soft tissue neoplasms. This analysis suggested a diagnosis of MGCT. Given the aggressive biology of the tumor, the patient was monitored closely with physical examinations and ultrasonographic monitoring of the percutaneous and scar region.

In August 2023, nine months post-surgery, a new nodule was detected at the previous surgical scar on the right chest wall. The nodule was firm, non-mobile, and consistent with previous findings. Chest wall ultrasonography revealed a hypoechogenic, subcutaneous, suprafascial nodular formation approximately 18 mm in diameter, suggestive of recurrent neoplastic disease.

In September 2023, an excision of an 8 cm x 2.5 cm skin lozenge from the right submammary site was performed, centered on the surgical scar. Pathological examination revealed skin with dermal scar fibrosis, without any residual neoplastic tissue.

**Figure 3:**
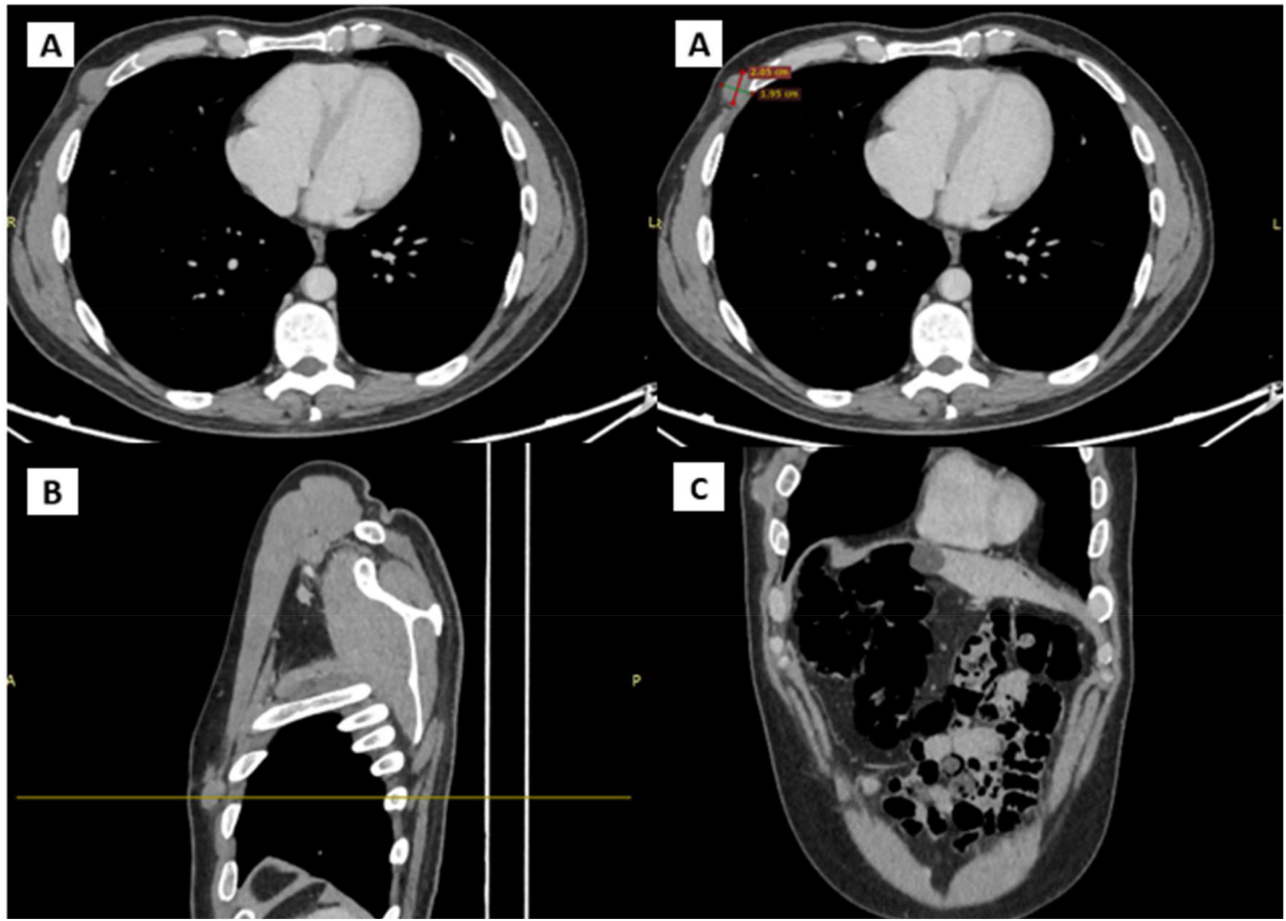
CT scans showing the presence and anatomical relation of the heteroplastic mass. **(A)** Transverse plane **(B)** Sagittal plane **(C)** Coronal plane.

In January 2024, a new nodule was identified at the previous surgical site, resembling prior findings. Ultrasound showed a hypoechogenic, subcutaneous, suprafascial nodular formation approximately 25 mm in diameter, indicative of likely recurrent neoplastic process. A CT scan with contrast (**Figure 3**) on the same day revealed a 20 mm nodular formation in the QIE of the right breast, in the context of subcutaneous fat, showing inhomogeneous enhancement and adherence to the intercostal muscles and the anterior margin of the right fifth rib, consistent with the known solid lesion from previous assessments.

**Figure 4:**
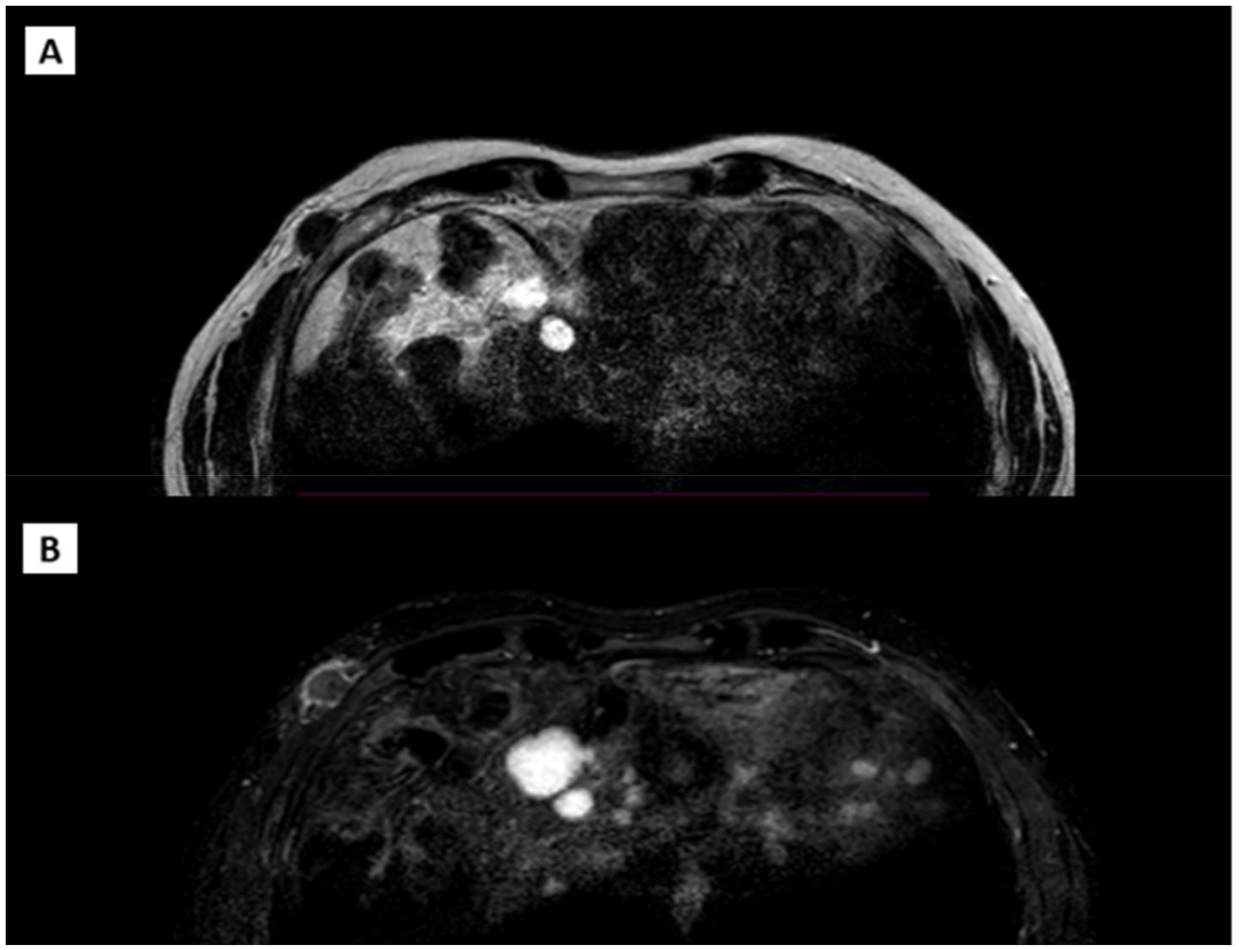
**(A)** T1 weighted acquisition. **(B)** T2 weighted acquisition.

The patient also underwent an MRI (**Figure 4**) of the right breast and chest wall with a paramagnetic contrast agent. The MRI revealed an irregular nodular formation, approximately 2.8 cm x 1.4 cm, in the right anterolateral subcutaneous region at the height of the fifth rib at the anterior inferior margin of the pectoralis major. The mass had an inhomogeneous signal, predominantly internal fluid, with intense peripheral enhancement and a soft tissue infiltrating appearance.

In April 2024, a major surgical intervention was performed under total anesthesia. The surgery removed a skin lozenge close to the right chest wall, measuring 6 cm x 2 cm and 5 cm thick. Histopathological examination revealed a nodular lesion with circumscribed margins of 2 cm x 1.3 cm, located in the hypodermal section of the operative specimen. The lesion bordered the deep margin and was largely contained within the other margins of the surgical excision. Microscopically, the morphological picture showed a nodular hypodermal neoplasm consisting of epitheliomorphic cells with characteristic clear cytoplasm, displaying a vaguely nodular solid growth pattern and an infiltrative attitude toward the muscle tissue. There was very little fibrous intercellular component. Immunohistochemical analysis showed positivity for S100, CD68, Inhibin, MITF, TFE3 and negativity for MELANA and CALRETININ. The Ki-67 proliferative index was between 5% and 7%. Histomorphology and immunohistochemical assessment confirmed the recurrence of GCT.

The short-term recurrence indicated aggressive biological behavior of the neoplasm. Genetic analyses using next-generation sequencing (NGS) technology did not reveal any relevant mutations sui for diagnostic characterization or therapeutic strategies.

Despite the major surgery, the patient did not experience significant post-operative complications and achieved a complete recovery. Additionally, there were no functional or aesthetic impairments. In June 2024, the patient underwent a skin and pericardial subcutaneous ultrasound with monitoring of axillary, laterocervical, submandibular, supraclavicular, and inguinal lymph node stations. The examination did not reveal any suspicious elements for disease recurrence.

Following multidisciplinary discussion, there was no indication for adjuvant radiotherapy treatment. The patient was then a candidate for close follow-up with physical esaem and ultrasonography of the scar and pericatricial region, abdomen, and axillary, supraclavicular, laterocervical, and inguinal lymph node stations.

## Discussion

Granular Cell Tumors (GCTs) are rare neoplasms first described by the Russian pathologist Alexei Abrikossoff in 1926. Typically benign, GCTs constitute a small percentage of all soft tissue tumors, and their exact cause remains a topic of debate. The most widely accepted hypothesis is that GCTs originate from Schwann cells or their precursors, supported by the strong and consistent positivity for S-100 protein, a marker for neural crest-derived cells. GCTs primarily affect adults, with a higher prevalence in females, typically diagnosed between the second and sixth decades of life. The male-to-female ratio ranges from 1:1.8 to 1:2.4, indicating a significant female predilection.

GCTs account for approximately 0.5% of all soft tissue tumors, with only about 1-2% being malignant. They most commonly occur in the head and neck region, particularly the tongue, which accounts for about 40% of cases. Other common locations include the breast (15%), respiratory tract (10%), and esophagus (2%).

The clinical presentation of GCTs varies depending on the tumor location. Most GCTs are asymptomatic and discovered incidentally. When symptoms occur, they may include a painless nodule or mass, discomfort, or functional impairment depending on the anatomical site involved (4). In rare cases, particularly in the gastrointestinal tract, GCTs can cause more serious symptoms like obstruction or bleeding. Diagnosis of GCTs is primarily based on histopathological examination following a biopsy. Imaging studies such as ultrasound, MRI, or CT scans can aid in assessing the tumor size, location, and extent, but are not definitive for diagnosis. Fine-needle aspiration (FNA) can sometimes be used, but its utility is limited due to the granular cell’s distinctive features being less apparent in cytological preparations.

The cytological features of GCTs can sometimes make differentiation from other tumors challenging. The cells are generally round to polygonal with abundant, finely granular, eosinophilic cytoplasm (5). The nuclei are round to oval, eccentrically placed, with fine chromatin and occasionally small, inconspicuous nucleoli. These cells typically have indistinct cell membranes and a granular background due to the accumulation of lysosomes and autophagosomes. Under electron microscopy, these granules can be observed along with replicated basal lamina. Immunoistochemically, both benign and malignant tumor cells typically stain positively for S-100, CD68, neuron-specific enolase, CD57, inhibin, calretinin, TFE3, SOX10, and nestin (6, 7).

Malignant GCTs are rare and tend to present as rapidly growing tumors, often larger than 5 cm. The diagnosis of malignant GCTs is based on specific histopathological criteria. According to studies by Fanburg-Smith et al. (5), a tumor is classified as malignant if it exhibits three or more of the following six features: 1) necrosis, 2) presence of spindle cells, 3) mitosis rate of more than 2 per 10 high-power fields, 4) presence of a vesicular nucleus with a large nucleolus, 5) high nucleus-to-cytoplasm (N) ratio, and 6) pleomorphism. If two of these features are present, the tumor is labeled as “atypical GCT.”

Whole-exome and targeted sequencing have identified recurrent inactivating mutations in ATP6AP1, ATP6AP2, and ATP6V0C in up to 72% of GCTs (8, 9). These mutations in Schwann cells, when replicated *in vitro*, not only triggered oncogenesis through increased phosphorylation of PDGFR-B, SFK, and STAT-5 but also led to impaired vesicle acidification, disrupted endocytosis, and the accumulation of intracytoplasmic granules. It’s also hypothesized that the nuclear localization and activation of MITF/TFE3, caused by V-ATPase dysfunction, might contribute to GCT development, as TFE3 gene fusions and the nuclear presence of resulting fusion proteins are seen in various sarcomas. These mutations, found in less than 0.1% of other cancers, are considered diagnostic of GCTs (8–10). The disruption of ATP6AP1, ATP6AP2, and ATPV0C impairs the V-ATPase (H+ ATPase) complex, reducing lysosomal activity. This disruption not only aligns with the typical cytoplasmic characteristics but also explains the tumor’s positive immunohistochemical staining for TFE3 and wild-type MITF, as lysosomal inhibition activates the transcription factors MITF, TFE3, and TFEB (8, 9).

Mutations in genes associated with the TGFβ and MAPK pathways have also been reported. Loss-of-function mutations in TGFBR1, TGFBR2, and LTBP2 can allow cells to evade the inhibitory effects of TGF-β, leading to increased cell proliferation. Additionally, a loss-of-function mutation in MAP3K15 has been identified, which is thought to promote tumor cell survival by preventing apoptosis. Both the TGFβ and MAPK pathways are crucial in regulating the cell cycle, tumor formation, and metastasis in many cancers. While these pathways typically have opposing roles TGFβ inhibits cell proliferation, whereas MAPK promotes it in the context of malignant transformation, they appear to support each other, with TGFβ shifting from a tumor suppressor to a promoter of metastasis (11).

The PIK3CA gene encodes the catalytic subunit of phosphatidylinositol-3 kinase (PI3K), which is activated by various tyrosine kinase receptors, including EGFR, ERBB2 (HER2), RET, MET, and VEGFR. PI3K then initiates downstream AKT/mTOR signaling, which promotes cell survival, proliferation, growth, and motility. In malignant GCTs, alterations in key oncogenes such as TP53 and PIK3CA have been observed, whereas these genes remain unaltered in benign GCTs (10).

GCTs also express markers such as CD63, LC3 (a specific marker of autophagy), and the antigen-presenting cell marker HLA-DR. Strong immunoreactivity for HLA-DR and CD68 suggests that GCTs may exhibit an antigen-presenting cell (APC) phenotype. HLA-DR is typically expressed by APCs (like monocytes, macrophages, and dendritic cells), B lymphocytes, and activated T lymphocytes. The significance of these findings is still debated. It’s possible that, in addition to CD68, HLA-DR immunoreactivity might be reactive and play a role in antigen presentation during the innate immune response in GCTs. Alternatively, HLA-DR expression could be a positive prognostic marker for survival, or it might indicate epithelial–mesenchymal transformation, suggesting tissue dedifferentiation rather than involvement in tumor antigen presentation (12).

Understanding these molecular alterations is crucial for advancing research and developing systemic therapies.

The mainstay of treatment for GCTs is surgical excision with clear margins to prevent recurrence. Complete excision is generally curative for benign GCTs. Malignant GCTs, which are exceedingly rare, require more aggressive treatment, including wider surgical resection, and may benefit from adjuvant radiotherapy or chemotherapy. The prognosis for benign GCTs is excellent, with a very low recurrence rate when adequately excised. Malignant GCTs, however, have a poorer prognosis, with higher rates of recurrence and metastasis (13).

For recurrent cases, additional surgery may be necessary. Close follow-up is essential to monitor for recurrence, particularly in cases where the initial excision was incomplete. Radiation therapy is generally not used for benign GCTs due to their indolent nature. However, it may be considered for malignant GCTs, especially when surgical margins are positive, or the tumor is unresectable. The use of adjuvant radiotherapy (dose not specified) after local excision of a large (5 cm) GCT has also been reported, with no evidence of recurrence or metastasis at 5 years (14). However, further investigation will be needed in order to better define the role of radiotherapy in the management of GCT.

The efficacy of classic cytotoxic chemotherapy in clinically malignant GCTs seems very limited, based on the published evidence, with few cases reported in the literature achieving a notorious dimensional response (15, 16). In literature are described sporadic cases of response to gemcitabine plus paclitaxel (6) and carboplatin plus etoposide (16, 17). Metastatic GCTs are relatively chemo-resistant; however, there is growing evidence of the benefit of using pazopanib and other targeted therapies in this histology (18).

Alterations in the MAPK (10, 11), PI3K (10), TGFβ (10, 11) pathways, and the description of VATPase (8, 9) variants are considered promising therapeutic targets that may provide novelties in the near future.

This case underscores the malignant potential and aggressive biological behavior of GCTs. The patient required multiple surgical interventions due to recurrent disease. Despite the generally benign nature of most GCTs, this case of MGCT demonstrated a propensity for rapid recurrence and infiltrative growth, posing significant challenges in management. The aggressive course of this MGCT highlights the necessity for vigilant long-term follow-up and comprehensive management strategies, including the potential use of advanced imaging techniques and possibly adjuvant therapies, even though they are not conventionally applied in benign cases. Genetic analyses, although not revealing actionable mutations in this instance, may become increasingly relevant as our understanding of the molecular underpinnings of GCTs evolves.

## Conclusion and patient perspective

In conclusion, this case exemplifies the complexity of diagnosing and treating MGCTs, emphasizing the need for a multidisciplinary approach and the consideration of more aggressive therapeutic strategies to manage such rare but potentially life-threatening neoplasms. Continued research and clinical awareness are essential to improve outcomes for patients with MGCT. Considering the numerous recurrences and the biological aggressiveness of the tumor, the patient was a candidate for restricted follow-up with physical examination and ultrasound of the lymph node stations and scar area. The patient was followed up at a reference center for this type of pathology and due to the complexity and rarity of the diagnosis, two histological revisions were performed. Due to the numerous surgeries received, the patient had to change employment due to reduced mobility of the right upper limb resulting from the surgery. The patient will continue close clinical and instrumental follow-up.

## Data Availability

The raw data supporting the conclusions of this article will be made available by the authors, without undue reservation.
